# Droplet Transportation through an Orifice on Electrode for Digital Microfluidics Modulations

**DOI:** 10.3390/mi12111385

**Published:** 2021-11-12

**Authors:** Ting-Chia Chu, Yen-Wen Lu

**Affiliations:** Department of Biomechatronics Engineering, National Taiwan University, Taipei 10607, Taiwan; r07631024@ntu.edu.tw

**Keywords:** chip-to-chip interface, electrowetting on dielectric (EWOD), water–oil separation, orifice, paper microfluidics

## Abstract

A digital microfluidic modular interface (chip-to-chip interface) which possesses an electrode with an orifice to vertically transport core–shell droplets is presented. The electrodes were geometrically designed to promote droplet deformation and suspension. The droplets were then applied with an electrical potential for insertion into and passage through the orifice. The concepts were tested with three types of droplets at the volume of 0.75~1.5 μL, which is usually difficult to transfer through an orifice. The integration of electrowetting on dielectric (EWOD) with paper-based microfluidics was demonstrated: the droplet could be transported within 10 s. More importantly, most of the core droplet (~97%) was extracted and passed through with only minimal shell droplets accompanying it.

## 1. Introduction

Electrowetting on dielectric (EWOD) has recently gained attention because of its space flexibility and route programmability for fluid manipulation, compared to traditional channel-based microfluidics [1,2,3,4,5]. EWOD, along with dielectrophoresis (DEP), can result in electromechanical forces to transport droplets or liquids with particles [6,7]. EWOD, without the need for pumps or valves, can freely move fluids as droplets with pathways capable of being re-programmed to perform a series of biochemical reactions. EWOD has been implemented for a wide range of biochemistry and Lab-on-a-Chip (or LoC) applications by integration with functional components such as photosensors, microheaters, electrochemical sensors, and mass spectrometers [8,9,10,11].

However, incorporating multiple functions into a single LoC device still presents a great challenge in microfluidics or EWOD techniques [12]. For instance, an EWOD chip was coupled to nanoelectrospray ionization mass spectrometry (nESI-MS), and it consisted of a capillary emitter between the top and bottom ITO plates; the chip was applied for dried blood spot (DBS) analysis for detecting hepatorenal tyrosinemia [8]. A green LED and a photodiode were integrated into an EWOD chip for glucose measurement in human physiological fluid [9]. An electrochemical immune assay with an array of electrodes was constructed on an EWOD chip [10]. Although these examples show the promising, broad applications of EWOD chips, they are constrained by their original designs to have a limited number of reagents, samples, or functions. A more practical method is not just to have multiple components on a single chip but to integrate multiple modules. The interface that connects different modules for EWOD chips, therefore, is vital.

A few examples have been shown by transporting a droplet on two different modules. S.-K. Fan et al. designed a modular interface with two gaps between overlapping plates; water–air droplets at 1.5~3.5 μL were transported chip-to-chip against gravity at 105 mm/s by sequentially switching the 136 driving electrodes [13]. H Feng et al. aligned chips with different gap widths and thicknesses, in parallel, as an interface to transfer droplets at 5~15 μL [12]. W. Wang et al. employed force balance analysis to reveal the conditions for water–air droplets to cross the open/closed boundary: water droplets can be easily moved, while oil droplets require additional DEP actuation and oleophobic treatment on the electrodes [14]. These modular interfaces possess the characteristics of simple alignment and minimum interfacial space, but the droplets are restricted to lateral movements (e.g., 2D). A chip-to-chip interface that allows vertical droplet movements (e.g., 3D) is preferred, not only to minimize the space but also to reduce the dead volume.

Although a 3D EWOD chip-to-chip interface has been shown in the open EWOD configuration to transport the droplet between two plates [15], it is preferable to have the interface in the closed EWOD configuration, which is commonly adapted in practical applications [16]. Bander et al. introduced an orifice in the middle of the electrode to transport a 3 μL core–shell droplet in the closed EWOD configuration for a 3D chip-to-chip interface [17]. However, examples are often applicable to large droplets (e.g., 2.5 to 15 μL), compared to smaller droplets, in EWOD configurations. Larger droplets usually have gravitational flattening [18] to promote their transportation across the gap [9,19,20,21]. Since their characteristic lengths are larger than the capillary lengths, a few of them utilize small droplets (e.g., <1.5 μL), which are difficult to effectively transport between chips.

We propose a module interface to vertically transport a droplet through an orifice in an EWOD chip for three types of small droplets: (i) water, (ii) water–oil core–shell, and (iii) (water + surfactant)–oil core–shell droplets, in air. An orifice on the electrode with the designed geometry serves as the vertical pathway between modules for the 3D chip-to-chip interface. The electrode geometry deforms the droplets, ready for insertion into the orifice. Possible applications of the module interfaces are demonstrated with the integration of EWOD and paper-based microfluidics, which is tested and evaluated.

## 2. Material and Methods

### 2.1. Device Fabrication

The EWOD chip fabrication started with the lithography technique. The electrodes were patterned on indium tin oxide (ITO) glass (7 Ω/sq, Ruilong Inc., Taipei, Taiwan). The ITO glass was cleansed with acetone and methanol under the vibration of an ultrasonic cleaner for 5 min and then baked at 115 °C for 2 min for dehydration. The positive photoresist S1813 (MicroChem Corp. Westborough, MA, USA) was dispensed and spun at 1000 rpm for 20 s, and then at 4000 rpm for 60 s. The thickness of S1813 was 2 μm. The ITO glass was soft baked at 115 °C for 5 min. It was exposed for 10 s to provide a sufficient dose of energy with UV light of 365 nm wavelength. The ITO glass was then immersed in TMAH at 2.38% (MicroChem Corp., Taipei, Taiwan) for 1 min. The hard bake was completed at 140 °C for 10 min. The sample was immersed in HCl (Taiwan Maxwave Co., Taipei, Taiwan) for 80 s at 48 °C to pattern the ITO. Acetone was used to remove the S1813 after the etching process. An orifice was then drilled into the surface of the ITO glass (diamond bur with a diameter ranging from 0.3 to 1.1 mm and 11 mm in length from Jin Bao Shan Jewelry & Tool, Taipei, Taiwan) by a drilling machine (LT-848 from Tun-Hwa Electronic Material Co., Ltd., Taichung, Taiwan) at 10,000 RPM.

A dielectric layer consisting of 3 μm of Parylene-C was deposited by chemical vapor deposition (La Chi Enterprise Co., LH 300, Taipei, Taiwan) on the patterned ITO glass. A layer of Teflon (AF 1600, DuPont, Wilmington, DE, USA) of 55 nm thickness was spin coated and post-baked on top of the Parylene-C to provide a hydrophobic layer. Please note that the orifice did not have the sidewalls coated with Teflon, meaning the orifice was relatively hydrophilic compared to the ITO surface [22].

### 2.2. EWOD Actuation

An AC voltage signal at 1 kHz frequency was generated by a function generator (SFG-2120, Good Will Instrument Co., Taipei, Taiwan), amplified by a voltage amplifier (A-304 High Voltage Amplifier, A.A. Lab Systems, Kennett Square, PA, USA), and set at 110 V_rms_ to the relay board (Winsuntek Technology, Hsinchu, Taiwan). A personal computer (PC) was used to send out the control signal through a Data Acquisition (DAQ) card (USB-6509, National Instrument, Austin, TX, USA) to the relays to control the AC signals on the corresponding electrodes of the EWOD chip.

Image analysis was performed by using ImageJ to estimate the surface area; thereby, the droplet volume could be calculated with the known gap thickness.

### 2.3. Core–Shell Droplets

An amount of 0.75~1.5 µL of deionized (DI) water droplets with 0.05% (*w*/*v*) Tween20 was employed in the experiments. The water droplets were surrounded by either an air or oil shell (hexadecane). The small volume (nL) of the oil shell provided a lower drag force to the water droplet than that in the oil bath, and this configuration had the advantages of lowering the droplet breakdown voltage, preventing evaporation [23], and preventing oil insertion into the orifice. Table 1 shows physical properties of droplet configurations used in the experiments.

### 2.4. Vertical EWOD Configuration

**Figure 1:**
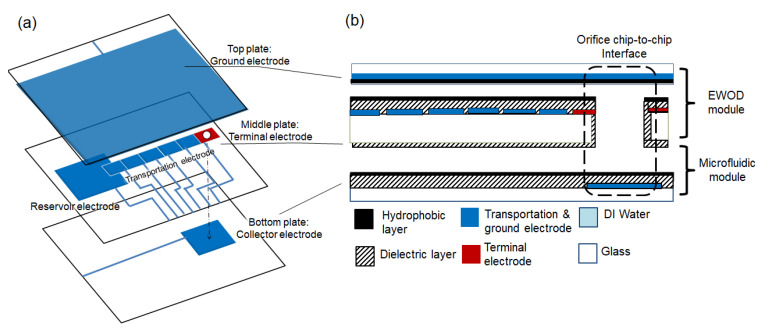
(**a**) 3D view and (**b**) cross-sectional view of the vertical EWOD configuration: A droplet can move vertically through the EWOD module. There are three plates: the top and middle plates form an EWOD module, while the bottom plate represents an external device. The top plate has ground electrodes. The middle plate has an array of electrodes with an orifice where the droplet can pass through. The bottom plate has a patterned electrode aligned to the orifice.

The experiments were performed by using the vertical EWOD configuration, as shown in Figure 1. The configuration had an EWOD module, formed by the top and middle glass plates, and a bottom glass plate. Three spacers (3M™, Taipei, Taiwan, Acrylic Adhesive 220 9502, 3M, Maplewood, MN, USA) were stacked and placed between the layers with a total thickness of 180 μm for the gap. An orifice 550 μm in diameter was drilled into the middle plate and served as the vertical pathway for the droplet as it vertically moved through the EWOD module. A micro-inspection zoom lens system (Optem^®^ Zoom 125C, Excelitas Technologies, Waltham, MA, USA) with a CCD camera (STC-620PWT., Omron Sentech, Carrollton, TX, USA) was used to observe the experiments.

To demonstrate the vertical functionality, the configuration was tested with the droplets in the EWOD module moving along the electrodes of the middle plate. The droplets were first dispensed onto the middle plate by a pipette (Gilson Pipetman P2L) and covered with the top plate. They were then transported to the terminal electrode. During the transportation, the top plate electrode was kept as a ground electrode (GND), while the terminal electrode and bottom plate electrode remained as an open circuit. Once the droplet arrived at the terminal electrode, it was inserted into the orifice and passed through. An actuation voltage was then applied to the electrode of the bottom plate to create a DC electric field and draw the droplet out of the orifice to the bottom plate.

## 3. Droplet Vertical Insertion

### 3.1. Three Stages

**Figure 2:**
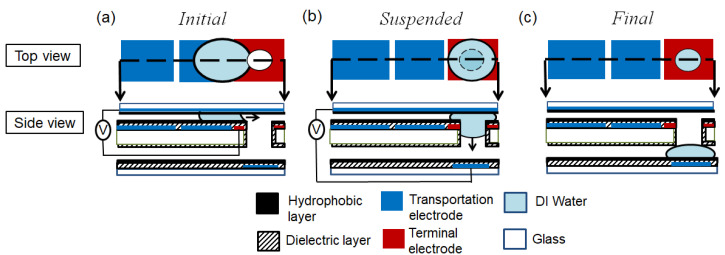
The droplet passes through (or inserts into) the orifice. There are three stages: (**a**) initial stage, when the droplet is transported to the terminal electrode by EWOD actuation; (**b**) suspended stage, when the droplet is inserted into the orifice at the terminal electrode; and (**c**) final stage, when the droplet is detached and vertically moves towards the electrode.

When the droplet vertically passed through (or was inserted into) the orifice, there were three stages: initial, suspended, and final, as shown in Figure 2. (a) In the initial stage, the droplet moved towards and arrived at the terminal electrode when the EWOD actuation voltages were applied. (b) In the suspended stage, the droplet spread around the orifice of the terminal electrode when no voltage was applied. (c) In the final stage, the droplet moved (or was inserted) into the orifice and passed through to the bottom plate when a voltage was applied to the electrode on the bottom plate.

### 3.2. Terminal Electrode

**Figure 3:**
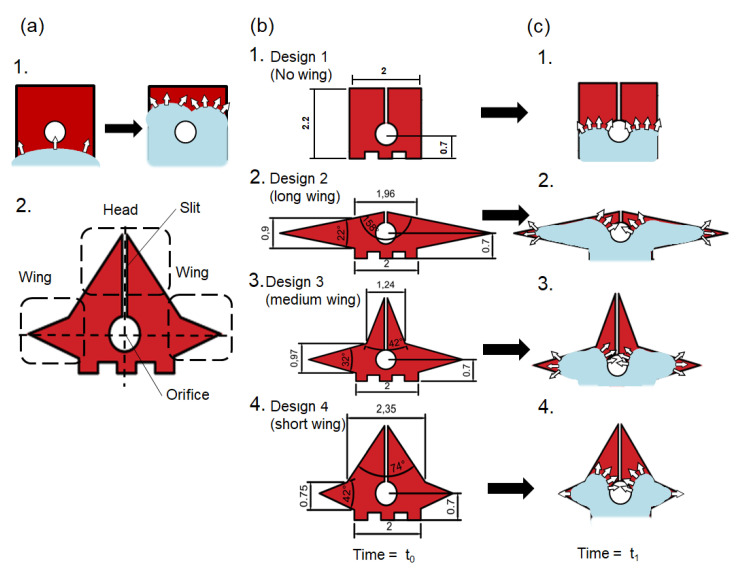
(**a-1**) The droplet spreads around the terminal electrode (square shape). (**a-2**) Geometrical definition of the terminal electrode. (**b**) The four designs of the terminal electrodes used in our study. (**c**) EWOD force directions at the droplet meniscus from the initial to the suspended stage on the terminal electrode.

The terminal electrode plays an important role in droplet insertion. The terminal electrode was designed to assist the droplet in moving into the orifice. The droplet first moved its contact line onto the terminal electrode; then, it split, circulated the orifice, and was pinned at the edge of the electrode [28], as shown in Figure 3(a-1). Although the EWOD forces kept pulling the droplet meniscus to deform around the orifice, the droplet did not have enough surface energy to overcome the energy barrier and move from the initial to the suspended stage.

To increase the surface energy of the droplet, our first improvement was to introduce a longer electrode perimeter on the terminal electrode. This allowed the droplet to deform longer contact lines before the suspended stage. The electrodes had wing and head parts, as shown in Figure 3(a-2), which allowed the droplet to spread around and circulate the orifice (i.e., clockwise and counter-clockwise). A slit design was also included in the head to establish an even longer perimeter and to prevent the droplet from merging before the suspended stage. It was also noted that the wing and head parts should not be too long, otherwise the droplet would deform along the electrode shape with the longer electrode, resulting in a longer time duration for the droplet when moving into the orifice.

Three different designs were used: (i) the terminal electrode with a short head and long wings; (ii) the terminal electrode with a medium head and wings; and (iii) the terminal electrode with a long head and short wings. All these designs had the same electrode areas (e.g., 4 mm^2^), with similar droplet volumes.

The wing parts kept the droplets from circulating the orifice quickly; they also slowed down the droplet motion towards the orifice. However, the head part had the EWOD forces to move the droplet meniscus—with some of the component forces—towards the orifice, and to help the droplet move into the suspended stage, as shown in Figure 3(c2-4). Design 2, therefore, had most of its droplet volume at the wing parts, while Designs 3 and 4 had relatively more droplet volume at the head part when time = t_1._

## 4. Capillary Length

Capillary length (λ*_c_*) is a length scaling factor that is related to surface tension and gravity forces according to [29], and it is defined as

(1) $$\lambda_{c}=\sqrt{\frac{\gamma }{\Delta \rho g}}$$

where γ is the surface tension, Δρ is the density difference, and g is the gravitational acceleration. When a droplet’s critical dimension is smaller than its capillary length (λ*_c_*), the surface tension dominates, and it mostly remains spherical [30,31]; otherwise, the droplet will be affected by gravity [18], and its contact lines will become susceptible to kinetic movement [32]. Designs 2 and 3, therefore, allowed the droplets to have larger critical dimensions and capillary lengths (λ*_c_*), making their contact lines prone to kinetic movements. The droplets were then suspended and ready for droplet insertion.

## 5. Results

### 5.1. Droplet Insertion

#### 5.1.1. Initial to Suspended Stage

**Figure 4:**
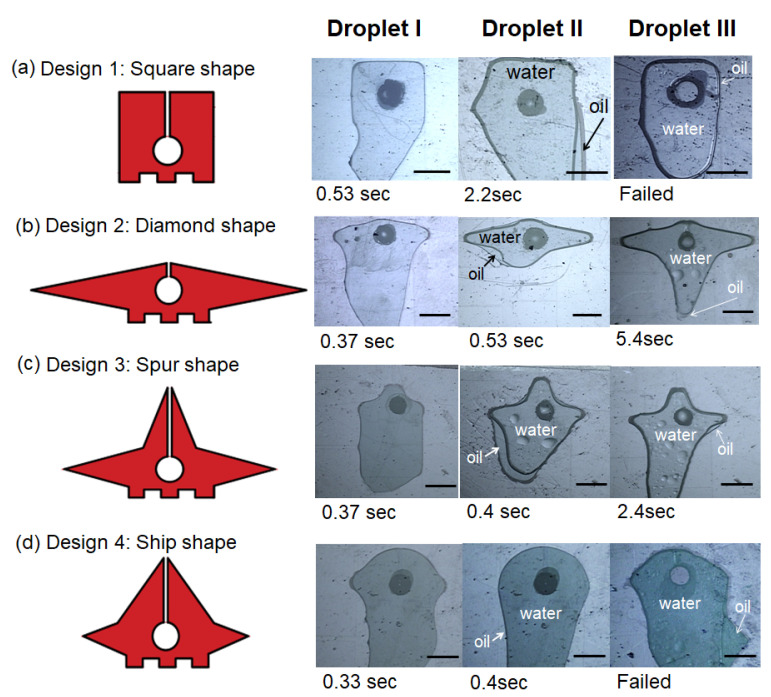
Droplets on the terminal electrodes of four designs moving from the initial to suspended stages (scale bar = 1 mm).

The first investigation was to realize the three stages in the droplet insertion. Droplets I, II, and III of 1 μL were created in the reservoir and moved to the terminal electrodes in four designs. Once the droplets arrived in the terminal electrode, they moved along the wings, towards the head of the electrode, and gradually towards the slit. The slit, set at 80 μm wide, prevented the droplets from fully surrounding the orifice and from randomly flowing around the orifice without entering the suspended stage. The droplets then suddenly spread and were suspended over the orifices, as shown in Figure 4. The sudden movement when the droplets entered the suspended stage caused a small disturbance in the droplet contact line, and this eventually made the contact line and the slit merge together.

Among the four terminal electrode designs, all three droplets used the longest time duration to reach the suspended stage on Design 1, shorter time durations on Designs 2 and 3, and the shortest time on Design 4. In general, the electrodes with the wing and head parts (e.g., Designs 2, 3, and 4) promoted the droplets to enter the orifice and reach the suspended stage easily, but the electrode without the wing or head parts (e.g., Design 1) had difficulty suspending the droplets.

The droplet contents also affected the droplet insertion. It was found that Droplet I used the shortest amount of time (e.g., 0.33~0.53 s) to reach the suspended stage. Droplets II and III, whose oil shells tended to stay on the relatively lipophobic surface of the middle plates, took longer to reach the suspended stage. In particular, Droplet III—a water droplet with a surfactant and oil shell—could only reach the suspended stage on Designs 2 and 3 and failed to reach the suspended stage on Designs 1 and 4.

Because not all of the droplets were suspended on Designs 1 and 4, the droplets were mainly manipulated on Designs 2 and 3 in the following experiments.

#### 5.1.2. Suspended to Final Stage

Until the electrical potential was applied on the electrodes of the bottom plates and the electric field was formed, the droplets remained suspended in the orifices and did not reach the bottom plate. In other words, the droplet on our vertical-transported device did not contact the electrode on the bottom plate, meaning it could not be vertically transported onto the electrode on the bottom plate via electrowetting forces. Instead, the droplet was attracted by the electric field, applied from a distance (~180 μm). However, the droplet on another planar-transported device [13] partially contacted its neighbor electrode, meaning it could move to the next electrodes via electrowetting forces.

**Figure 5:**
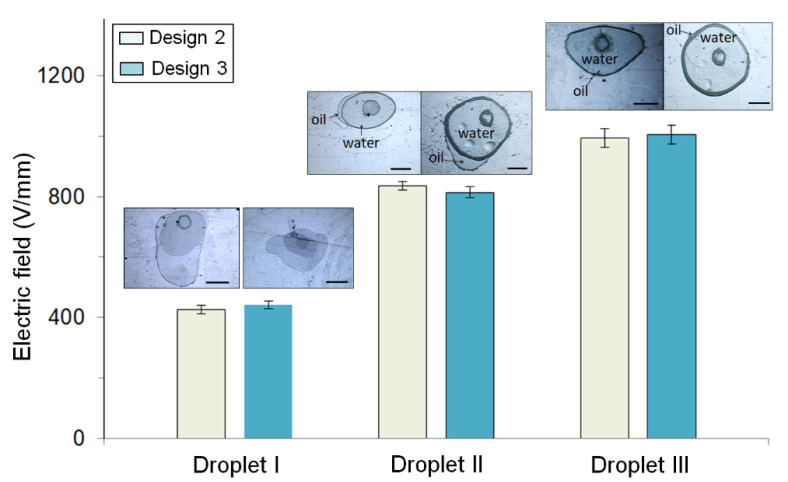
The minimal electric field required to move the droplet from the suspended to the final stage for droplet insertion. The images were taken when the droplets were inserted into the orifices and moved onto the bottom plates. As the droplets could not complete the droplet insertions on some of the terminal electrode designs, only the results from Designs 2 and 3 are shown (scale bar = 1 mm).

The minimum electric fields to transport the droplets on different terminal electrodes from the suspended to final stages were recorded and are shown in Figure 5. It was found that Droplet I entered the final stage at 429 V/mm on Design 2, and at 440 V/mm on Design 3; Droplet II at 835 V/mm on Design 2, and at 819 V/mm on Design 3; Droplet III at 994 V/mm on Design 2, and at 1006 V/mm on Design 3. Every droplet required its voltage at similar values, even though different terminal electrodes were used. In other words, once the droplets were suspended and moved into the orifice, the minimal voltages required to transfer them onto the bottom plates were similar. The geometry or designs of the terminal electrodes play minor roles during the third stage of droplet insertion. In addition, Droplets II and III required slightly higher voltages, mainly because their oil shells tended to stay on the surface (e.g., relatively lipophilic) of the middle plate. Moreover, Droplet III on Designs 1 and 4 is not shown in Figure 5 since it could not be suspended.

### 5.2. Droplet Volume Change during Insertion

#### 5.2.1. Droplet Recovery

Most of the core droplets completed the insertion process, while some satellite droplets were generated and left at the EWOD module. To evaluate the droplet insertion process, the volume recovery ratio of the core droplet ($R_{c}$) is defined as

(2) $$R_{c}\left(\%\right)=\left(\frac{C}{C_{o}}\right)\times 100$$

where $C_{o}$ and C are the volumes of the core droplets on the middle plate before the insertion, and on the bottom plate after the insertion, respectively.

**Figure 6:**
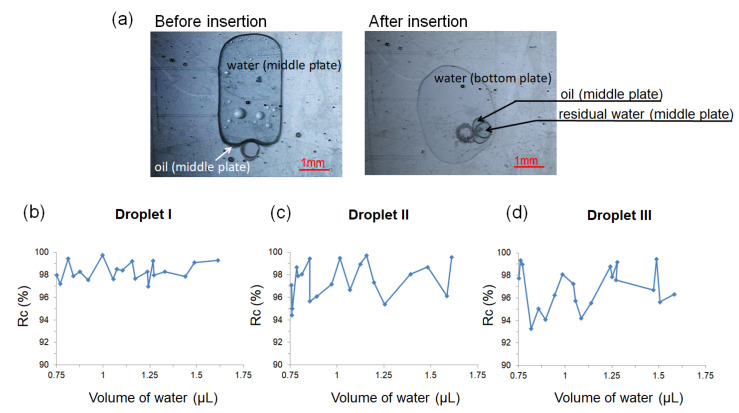
(**a**) Images of Droplet III before and after insertion. After insertion, residual water and most of the oil remained on the middle plate, while the majority of the water was on the bottom plate. (**b**–**d**) The recovery volume ratio of the core droplets for (**b**) Droplet I (water in air), (**c**) Droplet II (water–oil core–shell), and (**d**) Droplet III ((water + surfactant)–oil core–shell).

Figure 6 shows the droplet volume change and recovery ratio ($R_{c}$) for all three types of droplets (*C_o_* = 0.75~1.5 μL) before and after insertion (Figure 6a) for at least 20 attempts with the droplet volume ranging from small (0.75 μL) to large (1.5 μL). The average $R_{c}$ for Droplet I was 98.3% (standard deviation (std) = 0.8%); for Droplet II, it was 97.6% (std = 1.6%); and for Droplet III, it was 96.9% (std = 1.8%). The volume recovery ratio was different between the droplet types, but it had similar values for Designs 2 or 3. In other words, the droplets on the same terminal electrode designs had a similar percentage of the core droplets transferred from the middle onto the bottom plates. Additionally, Droplets II and III had a slightly lower R*_c_* after the insertion, mainly due to the drag force from the oil shell of the droplet.

#### 5.2.2. Droplet Shell Removal

During the droplet insertion, the majority of the core droplet (mainly water) was transferred onto the bottom plate, with very few portions of the water core remaining on the middle plate. Meanwhile, the oil (mainly hexadecane) stayed on the lipophilic Teflon surface of the middle plate rather than on the sidewall surfaces of the orifice [33]. In addition, the electrostatic forces were small due to the electric field from bottom plates during the insertion process, with only a small portion of hexadecane traveling with the water core droplet through the orifice.

The droplet oil shell removal ratio (R*_s_*) is defined as

(3) $$R_{s}\left(\%\right)=\left(1-\frac{S}{S_{o}}\right)\times 100$$

where *S_o_* and *S* are the oil volume in the oil–water droplet on the middle plate (EWOD module) before and after droplet insertion, respectively.

Over 20 tests, the average $R_{s}$ was 79.8% (std = 6.5%) for Droplet II; the average $R_{s}$ was 74.6% (std = 8.8%) for Droplet III. Droplet III had a smaller $R_{s}$ than Droplet II because it had an oil shell that tended to remain, owing to the presence of the surfactant. Finally, although a portion of the oil shell remained on the middle plate and could not be transferred onto the bottom plate, it usually evaporated in a few seconds. No additional heater was needed to remove the oil shell. Therefore, our proposed configuration effectively promoted the insertion and extraction of the core–shell droplet through the orifice, without the oil shell either on the middle or bottom plates.

## 6. Application Demonstration

**Figure 7:**
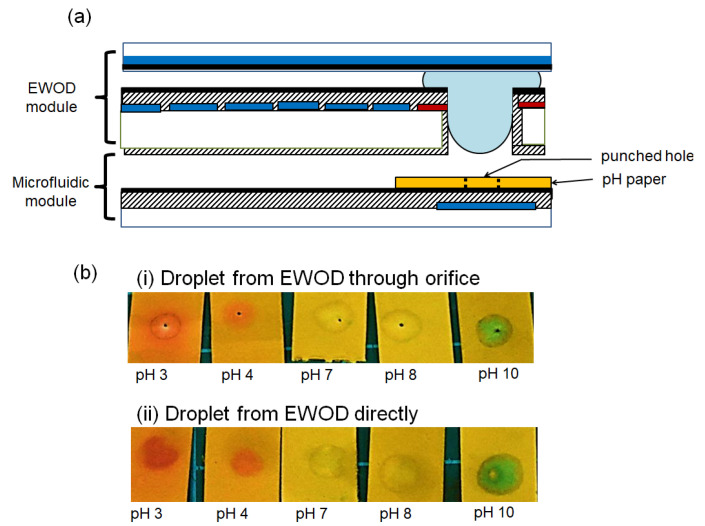
(**a**) Configuration of an EWOD device with pH paper. The pH paper has a punched hole, aligned to the orifice. (**b**) The results of transferring core–shell droplets on an EWOD device to pH paper: (**i**) The core–shell droplets are transferred through the orifice. Only some of the oil shell is transferred onto the pH paper with a minimal oil ring. (**ii**) The core–shell droplets are transferred directly (without the orifice). More of the oil shell is transferred onto the pH paper with an obvious oil stain.

To show the potential applications of droplet insertion, an aqueous droplet with different pH values was tested in an EWOD device with Designs 2 and 3 for the terminal electrodes with a pH test paper. A droplet of HCl (or NaOH) with a hexadecane oil shell was placed on the EWOD module, diluted with another DI water droplet, and transported to the terminal electrode ready for droplet insertion, followed by a pH value paper-based detection, as shown in Figure 7a. This process represents a common microfluidic manipulation and sample preparation for downstream diagnosis. The pH paper was punched with a hole so the electric field could be applied between the EWOD and pH paper to attract the droplet for insertion.

The sample droplets on the EWOD device were transferred (i) through the orifice and (ii) directly onto the pH paper; the results on the pH paper are shown in Figure 7b. The core–shell droplets transferred from the EWOD device through the orifice showed clear circular pH color marks with minimal oil ring stains, while the droplets from the EWOD module transferred directly (without the orifice) had slightly blurred pH color marks with obvious oil ring stains. The oil ring could cause confusion or mislead the interpretation of the results. The EWOD device with the orifice design, therefore, not only promoted the water core droplet insertion between different modules but also minimized the oil shell transferred during the process.

Finally, our proposed EWOD modules with orifices could be used to integrate multiple modules with their distinct functions. The integrations could potentially perform a series of steps from the EWOD module to other microfluidic or sensing devices, which could expand the overall functionality from sample preparation to pretreatment to diagnosis. More importantly, the droplet insertion, based on our proposed design, could not only effectively extract the core droplet but also separate the oil shell: another great advantage of using core–shell droplets in on-chip diagnosis. Our design allowed the water –oil core–shell droplets in the EWOD chips to be processed first for sample preparation and mixing, and then transferred through to another detection module (e.g., test paper, lateral flow device, or spectrometer) without the oil shell for better result analysis and reading.

## 7. Conclusions

An EWOD device that had geometry-designed electrodes with an orifice was presented as a chip-to-chip interface for droplet insertion. The core–shell droplets at 0.75~1.5 μL were vertically transported through the orifice within 10 s. A total of 97% of the water core droplet was transferred, while 75% of the oil shell droplet remained on the EWOD electrode. The chip-to-chip interface was proven to integrate an EWOD device with a pH test paper, showing its potential applications in extracting and transferring core–shell droplets from the EWOD to other microfluidic or sensing devices for conducting biological processes and analysis.

## Figures and Tables

**Table 1:** Droplets used in this study (data from [24,25,26,27]).

	Configuration	γ_LG(L)_ (mN/m) (Liquid–Gas (Liquid))	γ_LS_ (mN/m) Liquid–Solid	*λ_c_* (mm)	Purpose
Droplet I	Water–air droplet	76	92	2.7	General Purpose
Droplet II	Core: Water Shell: oil (hexadecane)	36	92	3.2	Lower the surface tension/prevent evaporation
Droplet III	Core: water + Tween20 Shell: oil (hexadecane)	7	22	1.8	Much smaller surface tension/prevent evaporation
